# Native Mass Spectrometry Reveals Ligand-Specific Conformational Heterogeneity of the β_1_‑Adrenergic Receptor Using Nanobody Probes

**DOI:** 10.1021/acs.analchem.6c04571

**Published:** 2026-09-26

**Authors:** Yi-Quan Wang, Guan-Ting Lian, Yi-An Chen, Rong Chen, Peng-Yi Wu, Hsiang-Hui Peng, Hsin-Yung Yen

**Affiliations:** † Institute of Biological Chemistry, 71561 Academia Sinica, Taipei 115201, Taiwan; ‡ Institute of Biochemical Sciences, National Taiwan University, Taipei 106319, Taiwan; § Chemical Biology and Molecular Biophysics Program, Taiwan International Graduate Program, Academia Sinica, Taipei 115201, Taiwan

## Abstract

G protein-coupled receptors (GPCRs) are central physiological signal transducers and represent a primary class of therapeutic targets. While emerging evidence highlights a critical link between ligand-induced receptor conformations and pharmacological outcomes, the structural diversity dictating GPCR-G protein interface interactions remains incompletely understood. High-resolution structural studies reveal discrete, ligand-stabilized states, but these static snapshots do not fully capture the dynamic processes underlying GPCR activation and ligand efficacy. To address this, we established a native mass spectrometry (nMS) platform to probe ligand-induced conformational heterogeneity at the GPCR intracellular interface. Using the β1-adrenergic receptor (β_1_AR) as a model, we demonstrated a strong correlation between ligand efficacy and receptor complex formation with diverse active-state-specific nanobodies. Notably, leveraging the quantitative capacity of nMS, we uncovered distinct propensities of two highly similar nanobodies, Nb80 and Nb6B9, and showed that Nb6B9 engages weak-agonist-stabilized receptor states more effectively than Nb80. These interactions indicate that Nb6B9 recognizes ligand-specific conformations distinct from active states induced by full agonists. To gain structural insights, we utilized Nb6B9 chimeras with exchanged complementarity-determining regions (CDRs) and identified CDR1 and CDR3 as the primary drivers for ligand-specific recognition, implicating the involvement of receptor transmembrane motifs TM5 and TM6 in this conformational diversity. Finally, we demonstrated that nanobody-based nMS can distinguish receptor responses induced by the two salbutamol enantiomers, implying stereochemistry-dependent intracellular interface heterogeneity. Together, our study highlights the utility of nanobody-based nMS for evaluating the structural heterogeneity and dynamic landscapes of GPCRs.

## Graphical abstract

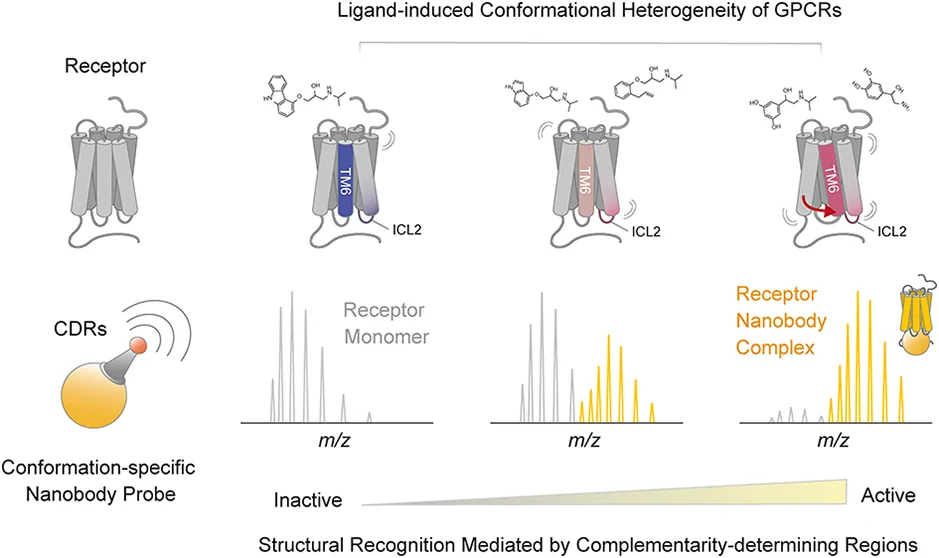

## Introduction

G protein-coupled receptors (GPCRs) regulate diverse signaling pathways by interacting with intracellular transducers, primarily G proteins and β-arrestins.^1,2^ Binding of orthosteric ligands induces conformational transitions within the receptor, remodeling its intracellular interface to accommodate specific transducers. Traditional cell-based functional assays, including CRE reporter and cAMP assays,^3,4^ assess ligand efficacy by measuring downstream signaling readouts and classify ligands as full agonists, partial agonists, antagonists, or inverse agonists. While these assays capture integrated responses shaped by receptor expression, transducer availability, signal amplification, and cellular context, they provide limited resolution of the molecular details underlying ligand-induced structural changes and how these changes modulate transducer recruitment, as critical determinants of drug efficacy and off-target effects.

High-resolution structural investigations using X-ray crystallography and cryogenic electron microscopy (cryo-EM) have provided unprecedented insights into GPCR architecture. Observations of discrete receptor conformational states, stabilized by diverse ligands and intracellular transducers, have mapped the structural signatures that define receptor activity.^5–10^ However, these static snapshots are often insufficient to explain how ligands with varying efficacies elicit distinct levels of transducer activation, particularly when only subtle structural differences are observed between receptor complexes bound to full versus partial agonists.^11,12^ Solution-based approaches, including nuclear magnetic resonance (NMR) spectroscopy and hydrogen–deuterium exchange mass spectrometry (HDX-MS), offer a dynamic perspective on receptor activation and suggest that ligand efficacy arises from shifting the equilibrium of GPCR conformational ensembles, rather than simply switching receptors between inactive and active states.^11–15^ Nevertheless, how these ligand-specific states drive differential transducer binding remains poorly understood, highlighting the need for novel approaches capable of delineating the structural heterogeneity of receptors engaged by diverse ligands.

Native mass spectrometry (nMS) has emerged as a powerful tool for probing the structure–function relationships of GPCRs. The high sensitivity and resolution of this technique enable direct quantification of receptor interactions with ligands, intracellular transducers, and allosteric environmental modulators such as zinc ions and lipids, thereby providing direct biophysical readouts of receptor activity.^16–21^ In parallel, nanobodies, recombinant antigen-binding fragments derived from camelid heavy-chain-only antibodies, have proven as highly effective surrogates for stabilizing specific active-state receptor conformations.^7,8,22,23^ Leveraging these synergistic tools, we developed a nanobody-based nMS platform to decode receptor conformational heterogeneity. By evaluating the interactions of the β1-adrenergic receptor (β_1_AR) with a panel of conformationally specific nanobodies using our nMS platform, we demonstrated their exquisite sensitivity in dissecting ligand-induced conformational diversity. Ligand-dependent receptor–nanobody complex formation broadly correlated with previously reported cAMP responses, providing validation of the quantitative nMS readout. Notably, we found that β_1_AR binding to Nb6B9 is markedly enhanced relative to its evolutionary precursor, Nb80, particularly when stabilized by partial and weak agonists. Complementarity-determining region (CDR) reversion experiments further identified distinct contributions of individual CDRs to Nb6B9 recognition, while comparison with available structures suggests potential contributions from receptor regions proximal to TM5, TM6, and ICL2. Finally, we revealed the differential responses of the salbutamol enantiomers as closely related stereoisomeric ligands, demonstrating the sensitivity of our platform in distinguishing receptor conformational states. Together, these results establish a streamlined framework that integrates quantitative nMS with conformation-selective nanobodies to interrogate ligand-specific conformational ensembles in GPCRs. By directly reporting ligand-specific receptor states through nanobody complex formation, our study highlights the potential of nanobodies as conformational probes that mirror intracellular transducer engagement.

## Results and Discussion

### Native MS Reveals Correlation between Nanobody-β_1_AR Complex Formation and Ligand Efficacy

Ligand-induced conformational changes reshape GPCR conformational ensembles toward active states, thereby governing the competency of transducer-mediated downstream signaling.^24–27^ To determine whether ligand-dependent receptor activation can be monitored through nanobody engagement, we optimized and purified a thermostabilized turkey β_1_AR in detergent micelles.^17,28,29^ Owing to the extensive functional characterization of this receptor, it provides an excellent opportunity to investigate the correlation between nanobody interaction and receptor activity using a well-defined ligand panel spanning a broad range of pharmacological efficacies.^28–30^

To probe ligand-dependent nanobody engagement, we established an nMS platform capable of capturing and quantifying complex formation between β_1_AR and nanobodies according to eq 1 (Experimental Section). Purified β_1_AR was buffer-exchanged into ammonium acetate containing G1, a nonionic oligoglycerol-based detergent suitable for nMS analysis of membrane proteins.^31^ To examine the correlation between nanobody complex formation and ligand efficacy (*E*_max_), we treated receptor with 100 μM ligand to ensure saturated occupancy across compounds with varying affinities,^30^ thereby minimizing occupancy-dependent variation (Figure S1 and Experimental Section). Ten ligands with distinct pharmacology profiles were examined, including full agonists (isoprenaline, formoterol, adrenaline, and noradrenaline), partial agonists (dobutamine, salbutamol and orciprenaline), weak agonists (pindolol) and antagonists (carvedilol and alprenolol) (Figure S2).

The resulting spectra revealed a clear ligand-dependent pattern of β_1_AR–nanobody complex formation. Full agonists, such as noradrenaline and adrenaline, promoted robust complex formation with all three active-state nanobodies (Nb80, Nb71, and Nb6B9),^7,8,23^ whereas partial agonists such as orciprenaline produced intermediate complex levels, and antagonists such as carvedilol resulted in minimal or undetectable complex formation (Figure 1a–c, Figures S2–S5 and Table S1). Conversely, the inactive-state-specific nanobody Nb60 exhibited weak complex formation under ligand-free or carazolol-treated conditions, which was further reduced by the full agonist isoprenaline, consistent with its preference for inactive receptor states (Figure S6a–c).

**Figure 1:**
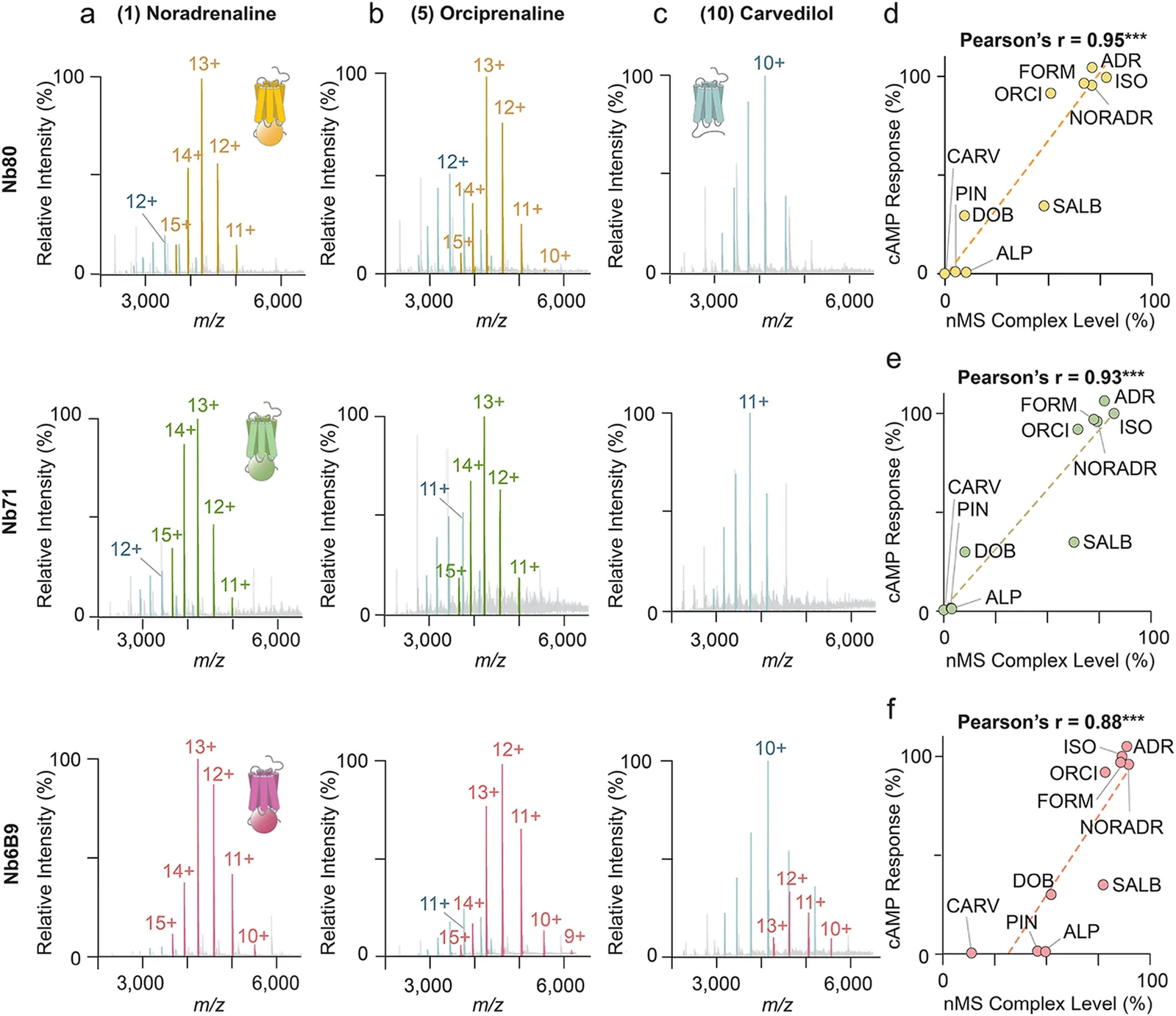
Native MS analysis of β_1_AR-nanobody complex formation and its correlation with ligand efficacy. (a–c) Representative nMS spectra of β_1_AR coincubated with Nb80, Nb71 and Nb6B9 in the presence of three different ligands: (a) (**1**) noradrenaline, (b) (**5**) orciprenaline, and (c) (**10**) carvedilol. Peaks corresponding to monomeric β_1_AR are colored in porcelain blue, while receptor-nanobody complexes are highlighted in yellow (Nb80), green (Nb71) and magenta (Nb6B9). (d–f) Correlation plots comparing cellular cAMP response (derived from literature data)^30^ against nMS-derived complex formation levels for (d) Nb80, (e) Nb71, and (f) Nb6B9. Statistical significance was determined by Pearson’s correlation coefficient (*r* values are indicated within each panel; *** *P* < 0.001). Data represent the mean values from three independent experiments.

To determine whether the quantitative nMS readout reflects ligand efficacy, we compared receptor-nanobody complex formation levels with previously reported cAMP signaling responses.^30^ Overall, significant positive correlations were observed across all active-state nanobodies (Pearson’s r values of 0.95, 0.93, and 0.88 for Nb80, Nb71, and Nb6B9, respectively) (Figure 1d–f), with a consistent trend observed for the engineered Gαs subunit, mini-Gs (Figure S7).^32,33^ These results suggest that ligand-dependent complex formation measured by nMS broadly reflects the reported β_1_AR signaling efficacy, supporting the use of active-state nanobodies as biophysical probes of ligand-dependent receptor activation.

### Nb6B9 Exhibits Distinct Ligand-Dependent Receptor Recognition

Comprehensive investigations of nanobody-receptor interactions revealed ligand-dependent recognition by Nb6B9. Compared with Nb80, its parental nanobody derived through directed evolution,^8^ Nb6B9 displayed substantially greater complex formation under selected weak-agonist and antagonist treatment, including alprenolol (Nb6B9, 49.6 ± 4.1%; Nb80, 10.4 ± 7.9%) and pindolol (Nb6B9, 45.7 ± 11.6%; Nb80, 5.2 ± 1.2%). By contrast, both Nb6B9 and Nb80 formed comparable complexes in response to full agonists such as isoprenaline (Nb6B9, 86.5 ± 7.8%; Nb80, 78.1 ± 2.3%) (Figure 2a and Table S1). This suggests that Nb6B9 possesses broader recognition capacity, engaging not only active conformations stabilized by full agonists but also receptor states induced by partial and weak agonists.

**Figure 2:**
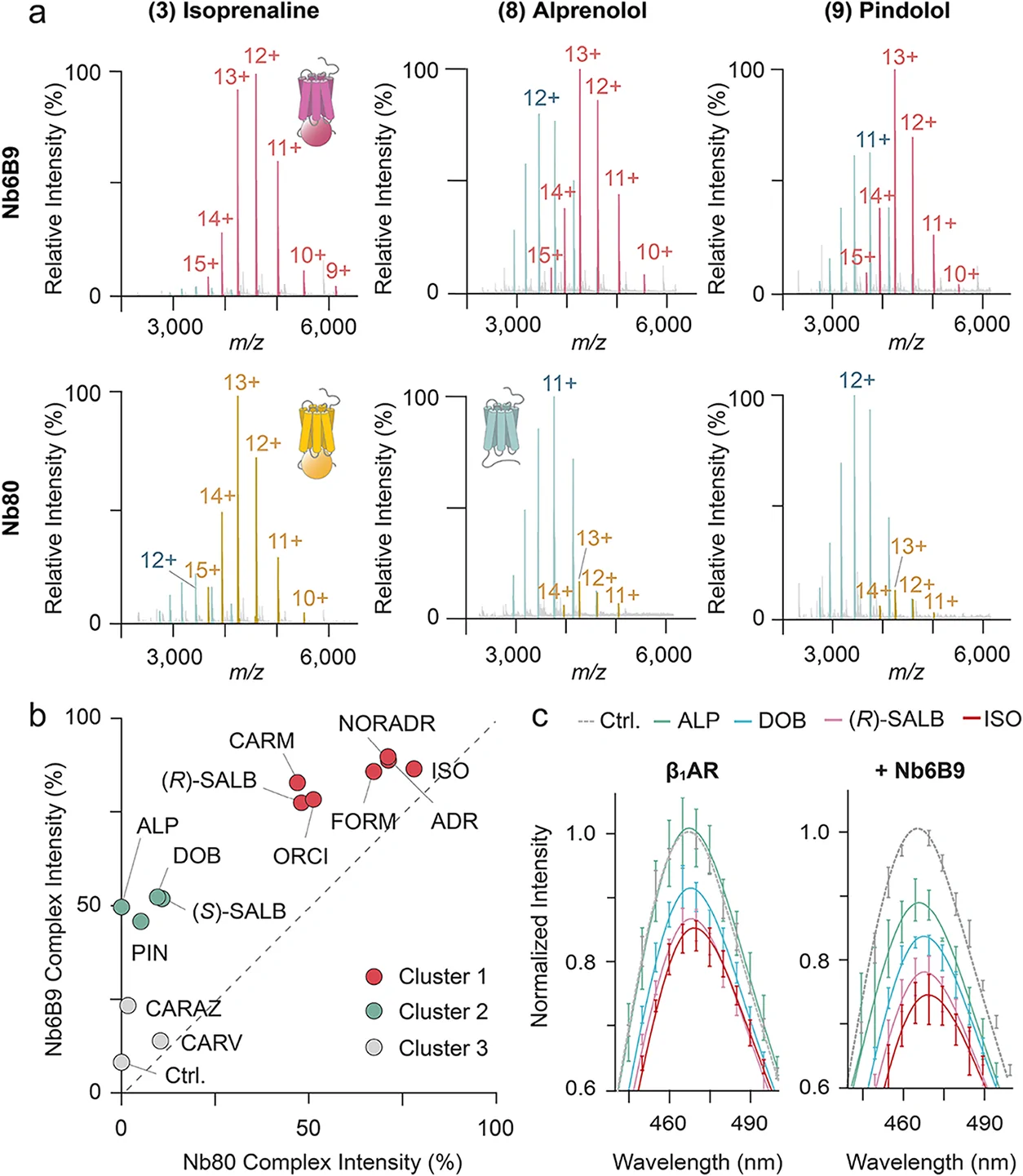
Ligand-specific β_1_AR conformations resolved by Nb6B9. (a) Representative nMS spectra of β_1_AR complex formation with Nb6B9 and Nb80 in the presence of (**3**) isoprenaline, (**8**) alprenolol, and (**9**) pindolol. The receptor-nanobody complexes are indicated in magenta (Nb6B9) and yellow (Nb80), whereas monomeric β_1_AR is shown in porcelain blue. (b) Scatter plot comparing the relative complex formation levels of β_1_AR bound to Nb6B9 versus Nb80 under each ligand treatment. The dashed diagonal denotes equal complex formation between the two nanobodies. Ligands are categorized into three distinct clusters based on their Nb6B9/Nb80 response ratios: red (Cluster 1, uniformly high response), green (Cluster 2, Nb6B9-preferential ligands with moderate response), and gray (Cluster 3, low response for both nanobodies). Data represent the mean values from at least three independent experiments. (c) Bimane fluorescence emission spectra of β_1_AR labeled at TM6 in the absence (β_1_AR alone) and presence of Nb6B9 under specified ligand treatments. Ligand-dependent shifts in fluorescence intensity and emission maxima indicate alterations in the local biophysical environment of TM6. Data represent mean ± SD from three independent experiments.

To further dissect nanobody-specific recognition, we performed nMS analysis across a panel of ligands (*n* = 13), including carmoterol, (*S*)-salbutamol, and carazolol in addition to the previous 10 ligands (Figure S2). We compared Nb6B9 with its parental nanobody Nb80, which shares 94% sequence identity (Figure S8a–b).^8^ The resulting profiles unveiled the distinctive responsiveness of Nb6B9 relative to Nb80. While both nanobodies exhibited robust complex formation with ligands known to stabilize active β_1_AR conformations, including noradrenaline, adrenaline, isoprenaline, carmoterol, formoterol, orciprenaline, and (*R*)-salbutamol (Figure 2b, cluster 1), a second cluster of ligands comprising dobutamine, pindolol, alprenolol, and (*S*)-salbutamol (Figure 2b, cluster 2) elicited approximately 5- to 9-fold greater complex formation with Nb6B9 than with Nb80, emphasizing the expanded recognition spectrum of Nb6B9. In contrast, the third cluster, including the vehicle control, carazolol, and carvedilol (Figure 2b, cluster 3), yielded low complex formation with both Nb80 and Nb6B9.

Previous studies have classified dobutamine as a β_1_AR partial agonist, whereas β-blockers such as pindolol and alprenolol exhibit promiscuous activity, acting as partial agonists, antagonists, or intrinsic sympathomimetic activity in a receptor- and assay-dependent manner.^34–37^ Our data suggest that Nb6B9 is capable of recognizing the receptor conformations stabilized by these ligands which were not specified in previous structures.^6^ Importantly, the lower complex formation observed for antagonists compared to agonists indicates that Nb6B9 still retains its selectivity toward active receptor, consistent with previous studies as an active-state-specific nanobody.^8^

To further investigate the intracellular conformations specifically recognized by Nb6B9, we employed a fluorescence assay to monitor receptor dynamics in solution. Monobromobimane (mBBr) was used to covalently label Cys^6.27^ at the cytoplasmic end of TM6 in β_1_AR (Figure S9a), providing a localized fluorescence readout of ligand-dependent structural rearrangements, a hallmark of GPCR activation.^38^ Native MS analysis confirmed successful bimane labeling, as evidenced by a clear ∼192 Da mass shift of the receptor corresponding to incorporation of a single bimane moiety (Figure S9b). In the absence of Nb6B9, isoprenaline treatment induced a prominent decrease in bimane fluorescence intensity, whereas partial agonists dobutamine and (*R*)-salbutamol elicited intermediate responses, and alprenolol was unable to produce a detectable fluorescence change (Figure 2c, left panel). These ligand-dependent fluorescence profiles indicate differences in the local environment of the TM6 reporter and are consistent with varying extents of TM6 rearrangement characteristic of full or partial activation, as previously described.^38^

To elucidate the structural impact of Nb6B9, we next coincubated the receptor with Nb6B9 following the same conditions described above. For the cluster 1 ligands such as isoprenaline and (*R*)-salbutamol, Nb6B9 further decreased the bimane fluorescence intensity, with the most prominent effect observed for isoprenaline. In contrast, cluster 2 ligands including dobutamine and alprenolol produced significant but more moderate decreases (Figure 2c, right panel). These findings align with the stabilizing effect of Nb6B9 on the active receptor^6,8^ and suggest that Nb6B9 can stabilize the alprenolol-induced configurations that are poorly recognized by mini-Gs and other nanobodies.

Together, our nMS and bimane data support a model in which ligands shift the β_1_AR intracellular conformational ensemble to varying extents. Potent agonists promote TM6 rearrangement that enables efficient recognition by mini-Gs, Nb71, Nb80 and Nb6B9. In contrast, weak agonists and sympathomimetic ligands could not induce sufficient TM6 displacement for robust complex formation with mini-Gs, Nb71 and Nb80. Notably, Nb6B9 retains the capacity to recognize and stabilize these atypical conformations, potentially distinct from canonical active states, thereby serving as a conformational probe that reveals weakly populated receptor states. More broadly, these findings underscore the utility of a nanobody-based nMS platform in distinguishing weak agonism from antagonism—pharmacological modalities that are poorly resolved by conventional signaling assays.

### Complementarity-Determining Regions Modulate Nb6B9-Specific Recognition

Conformational-selective enrichment through directed evolution yielded Nb6B9 with markedly enhanced affinity compared to its parental nanobody Nb80.^8^ Successive positive selections using the full agonist BI167107, combined with negative selections against the antagonist carazolol, introduced eight point mutations within the complementarity-determining regions (CDR1, CDR2, and CDR3) of Nb80 (Figure S8b).^8^ To investigate the molecular determinants underlying Nb6B9-specific recognition, we introduced a back-mutation strategy in which each CDR region of Nb6B9 was individually reverted to the corresponding Nb80 sequence. Three chimeric variants were generated: Nb6B9.1 (Nb6B9 carrying CDR1 substitutions A30S, L31I, and I33T), Nb6B9.2 (Nb6B9 carrying CDR2 substitution T56S), and Nb6B9.3 (Nb6B9 carrying CDR3 substitutions F100Y, I103 V, I104L, and D106E), each incorporating back mutations within the respective CDRs (Figure 3a). Native MS analysis of these variants against a ligand panel (Figures 3a–b,S10–S12, and Table S2) revealed that, overall, the CDR substitutions maintained high complex formation and had little effect on β_1_AR assembly in the presence of cluster 1 ligands such as isoprenaline, suggesting minimal impact of individual CDR mutations on recognition of the active receptor state (Figure 3a,3c). Notably, under cluster 2 ligand conditions, including (*S*)-salbutamol, dobutamine, pindolol, and alprenolol, reversion of CDR1 or CDR3 significantly reduced β_1_AR-nanobody complex formation by more than 25%, whereas these variants had no significant impact on cluster 3 ligands (Figure 3b–c). To gain structural insight into these CDR-dependent effects, we compared the structures of β_1_AR-nanobody complexes, including the isoprenaline-bound β_1_AR-Nb80 and the dobutamine-bound β_1_AR-Nb6B9.^6^ These structures revealed overall similar receptor-nanobody binding modes, with the CDR3 loop inserting deeply into the transducer-binding cavity and forming extensive interactions with TM5 and TM6, while the CDR1 loop contacts the intracellular surface near TM5 (Figure 3d). Given that CDR1 and CDR3 interact with receptor regions proximal to TM5 and TM6, we hypothesize that the configurations of these motifs are critical for Nb6B9-specific interactions.

**Figure 3:**
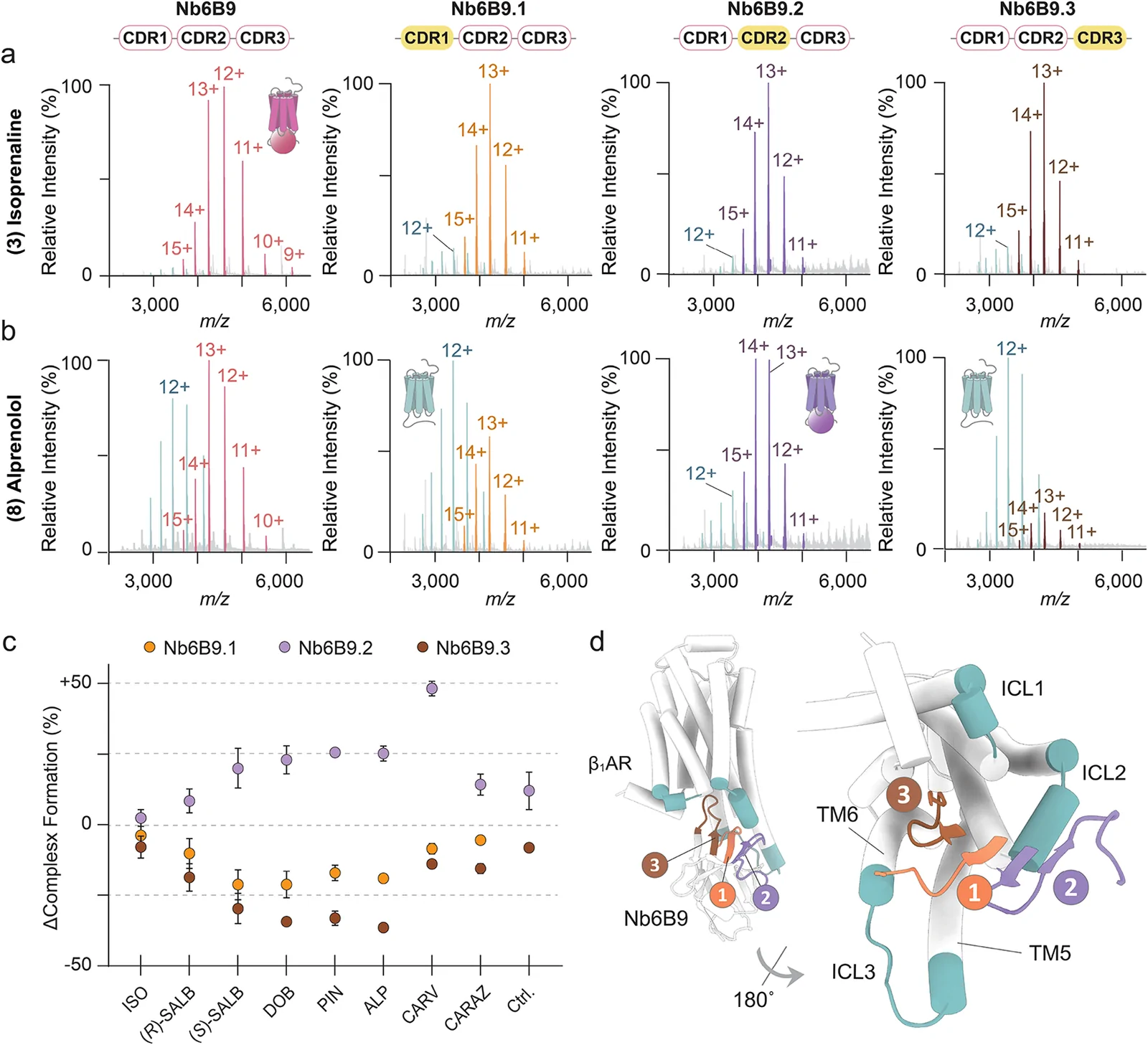
Complementarity-determining regions of Nb6B9 responsible for recognizing ligand-specific conformational states. (a–b) Representative nMS spectra of β_1_AR complex formation with Nb6B9 chimeric variants in the presence of (a) (**3**) isoprenaline and (b) (**8**) alprenolol. Chimeric constructs featuring the replacement of individual CDRs with corresponding Nb80 sequences are highlighted in yellow: Nb6B9.1 (CDR1 replacement), Nb6B9.2 (CDR2 replacement), and Nb6B9.3 (CDR3 replacement). Peaks corresponding to monomeric β_1_AR are shown in porcelain blue, and β_1_AR-nanobody complexes are indicated in magenta (Nb6B9), orange (Nb6B9.1), purple (Nb6B9.2), and brown (Nb6B9.3). (c) Comparison of complex formation levels between Nb6B9 chimeric variants and the wild-type Nb6B9 under various ligand treatments. Data represent the mean ± SD from three independent experiments. (d) Structural view of the Nb6B9-β_1_AR complex interface. CDR1, CDR2, and CDR3 of Nb6B9 are colored in orange, purple, and brown, respectively. The intracellular loops (ICL1, ICL2 and ICL3) of β_1_AR are shown in green.

Conversely, reversion of CDR2 markedly increased complex formation for cluster 2 ligands by approximately 25%, suggesting that an additional structural component underlies these nanobody-specific interactions (Figures 3b–c,S11, and Table S2). Given the spatial proximity of CDR2 to the ICL2 of the receptor (Figure 3d), we hypothesize that ligand-dependent differences in the local ICL2-proximal interface may contribute to CDR2-mediated recognition. This is supported by previous studies implicating ICL2 as a key determinant of receptor activation and transducer selectivity.^39–41^ Furthermore, a single-residue substitution from threonine to serine at position 56 (T56S) within CDR2 significantly enhanced nanobody binding in the presence of carvedilol, whereas carazolol did not elicit a comparable effect (Figure 3c). Because carvedilol is a well-characterized biased agonist that selectively stimulates arrestin signaling,^37,42,43^ these findings imply that CDR2 potentially contributes to the recognition of the local receptor conformation associated with arrestin recruitment. Collectively, our results demonstrate that Nb6B9 CDRs play distinct roles in discriminating ligand-specific β_1_AR conformational equilibria. While CDR1 and CDR3 expand recognition of active states stabilized by cluster 2 ligands, CDR2 preserves conformational selectivity through interactions with the receptor interface proximal to ICL2.

### Nanobody-Based nMS Analysis Differentiates β_1_AR Re sponses Induced by Salbutamol Enantiomers

To demonstrate the utility of nanobody-based nMS in distinguishing ligand-dependent receptor states, we examined the responses of β_1_AR to the two salbutamol enantiomers. Intriguingly, distinct engagement profiles were observed between the two chiral forms: (*R*)-salbutamol induced pronounced complex formation with both Nb80 and Nb6B9, whereas no detectable Nb80 assembly was observed with (*S*)-salbutamol (Figure S3 and Table S1). Notably, Nb6B9 displayed substantial complex formation in the presence of (*S*)-salbutamol, demonstrating its ability to recognize receptor conformations induced by the (*S*)-enantiomer (Figure 4a). To further quantify these stereoselective responses, we performed dose-dependent measurements of complex formation with these two compounds using Nb6B9 (Figure 4b and Table S3). Dose–response analysis with a variable-slope model according to eq 2 indicated that (*R*)-salbutamol (EC_50_ 9.1 μM, 95% CI: 6.6–13.3 μM) exhibited a significantly lower EC_50_ than (*S*)-salbutamol (EC_50_ 45.1 μM, 95% CI: 33.0–72.8 μM) for β_1_AR-Nb6B9 complex formation. In addition, (*R*)-salbutamol achieved a higher maximal response (*E*_max_ 82.6%, 95% CI: 75.3–99.1%) compared with (*S*)-salbutamol (*E*_max_ 55.7%, 95% CI: 48.2–73.8%) (Figure 4b). These results suggest that (*R*)-salbutamol induces more efficient formation of Nb6B9-compatible receptor states. In contrast, the lower complex-formation response of (*S*)-salbutamol implies that only a smaller fraction of the receptor ensemble adopts conformations compatible with Nb6B9 binding, even at high ligand concentrations. Collectively, these concentration-dependent profiles reveal differential receptor responses to the salbutamol enantiomers. Given that salbutamol is clinically administered as a racemic 1:1 mixture of its enantiomers, our findings support that (*R*)-salbutamol exhibits greater potency and efficacy than (*S*)-salbutamol.^44^

**Figure 4:**
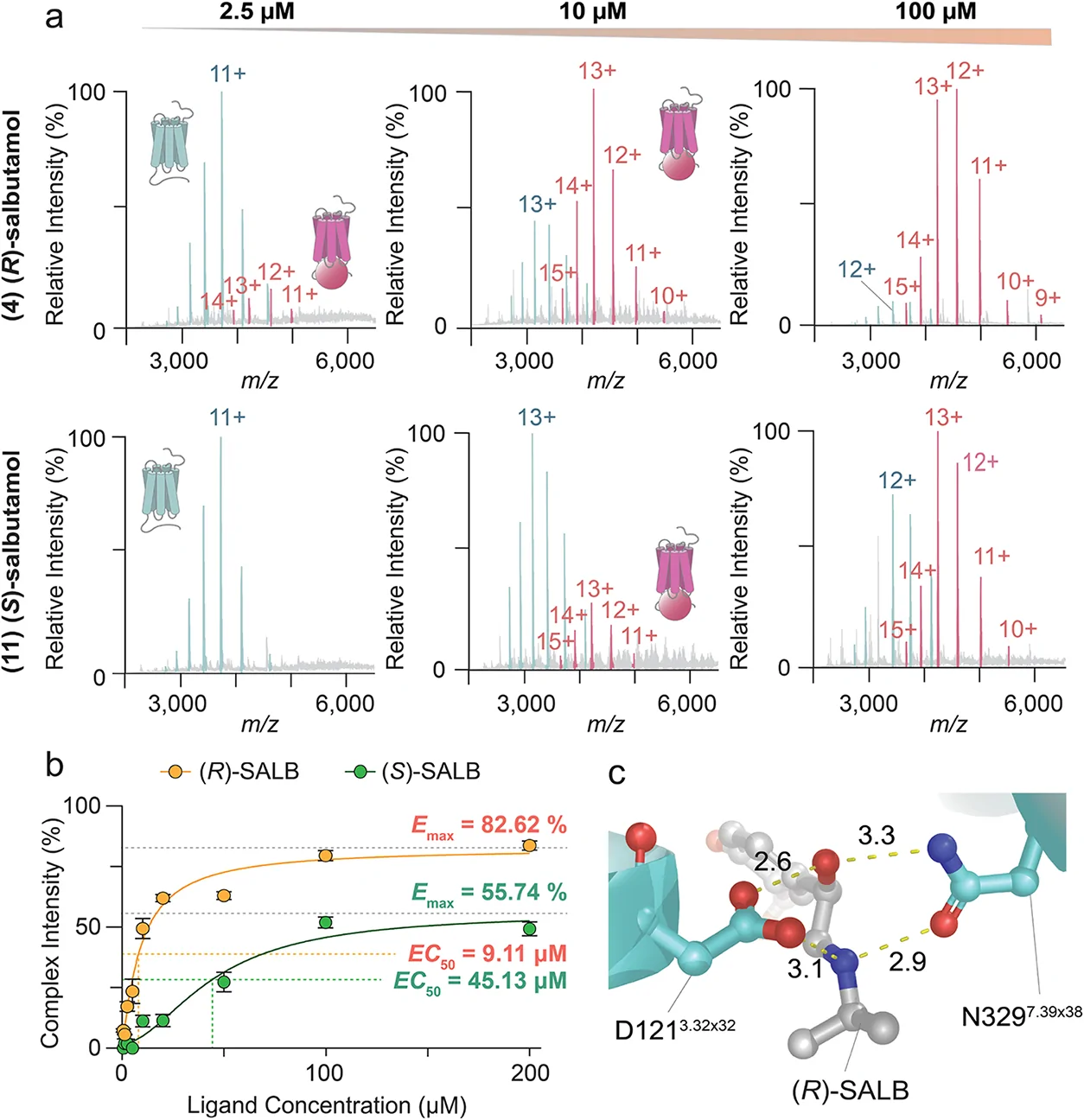
Stereochemistry-dependent β_1_AR conformations stabilized by a nanobody. (a) Representative nMS spectra of β_1_AR-Nb6B9 complex formation in the presence of (*R*)-salbutamol (top) and (*S*)-salbutamol (bottom) at increasing concentrations. Signals corresponding to monomeric β_1_AR are colored in porcelain blue, and the β_1_AR-Nb6B9 complex is depicted in magenta. (b) Dose–response curves for (*R*)- and (*S*)-salbutamol determined by nMS characterization. Nonlinear regression fitting using the variable-slope (four-parameter) model reveals distinct *E*_max_ and *EC*_50_ values (*R* ^2^ = 0.96 and 0.94, respectively). Data represent the mean with SD from three independent experiments. (c) Crystal structure of (*R*)-salbutamol (gray) bound within the orthosteric pocket of β_1_AR (cyan) (PDB: 6H7M). Key polar interactions between the β-hydroxyl group at the chiral center and surrounding residues are indicated by dashed lines, with distances labeled in angstroms (Å).

While conventional pharmacology largely attributes the activity of salbutamol to its (*R*)-enantiomer,^44^ our data support that (*S*)-salbutamol can elicit a measurable intracellular conformational response. To explore the structural basis of this stereochemistry regulation, we analyzed the previously reported (*R*)-salbutamol-β_1_AR-Nb6B9 ternary complex structure.^6^ In the orthosteric binding pocket, (*R*)-salbutamol is coordinated by a polar interaction network involving D121^3.32×32^ in TM3, S211^5.42×43^ and S215^5.46×46^ in TM5, and N329^7.39×38^ in TM7 (Figure S13a), using the combined Ballesteros–Weinstein/GPCRdb generic numbering scheme.^45^ In particular, the β-hydroxyl group of (*R*)-salbutamol is positioned to interact with D121^3.32×32^ and N329^7.39×38^, forming contacts of approximately 2.6–3.3 Å (Figures 4c andS13b), residues previously implicated in adrenergic ligand recognition and agonist-induced rearrangements of TM7.^28,45,46^ The inverted stereochemistry of (*S*)-salbutamol is expected to reposition this hydroxyl group away from the optimal interaction geometry, increasing the corresponding distances to approximately 3.6–5.3 Å relative to D121^3.32×32^ and N329^7.39×38^ (Figure S13b), potentially hindering structural propagation through TM7 to the intracellular receptor interface. Together, this finding highlights the sensitivity of nanobody-based nMS to uncover ligand-specific conformational responses that are associated with its downstream signaling.

## Conclusion

GPCR ligand efficacy is governed by conformational equilibria at the receptor intracellular interface. Owing to the heterogeneous and dynamic nature of these states, it remains challenging to define their connections with transducer engagement, a key determinant of the pharmacological outcomes. In this study, we established a nanobody-based nMS platform to quantitatively characterize ligand-dependent receptor recognition. By converting structural heterogeneity of β_1_AR into nanobody-recognition signals, we revealed a strong correlation between nanobody-receptor complex abundance and downstream signaling responses, providing a quantitative framework for profiling drug efficacy. This approach further demonstrated the differential β_1_AR responses induced by the two salbutamol enantiomers, highlighting the utility of nanobody-based nMS for resolving stereochemistry-dependent receptor recognition.

Notably, we uncovered a ligand-specific propensity of Nb6B9 which is distinct from other nanobodies. Structural analyses using bimane fluorescence measurements indicated that Nb6B9 stabilizes weakly populated conformational states of TM6 induced by weak agonists. CDR reversion experiments revealed critical contributions of CDR1 and CDR3 to Nb6B9-specific recognition. Additionally, CDR2 also contributed to ligand-dependent recognition, with structural comparisons suggesting possible involvement of the ICL2-proximal receptor interface. Given the high structural similarity between Nb6B9 and Nb80 in complex with β_1_AR, these findings provide detailed insight into the contributions of individual CDRs to receptor recognition. Collectively, this work underscores a new potential for the development of nanobodies in probing receptor conformational heterogeneity beyond the static snapshots captured by high-resolution structures. Future applications could extend this platform to other receptor types, full heterotrimeric G proteins, arrestins, and native lipid environments to investigate how cellular factors modulate receptor conformational landscapes. Integrating nMS with orthogonal biophysical techniques, such as fluorescence spectroscopy, NMR, HDX-MS, and structural modeling, could further clarify how ligand binding propagates from the orthosteric/allosteric pocket to the intracellular interface. Ultimately, our findings illustrate the potential of combining conformation-selective nanobodies with quantitative nMS to differentiate ligand-dependent receptor states within the GPCR system.

## Experimental Section

### Reagents and Ligands

Isoprenaline (I6504, ≥98%), epinephrine (E4642, ≥98%), dobutamine (D0676, ≥98%), pindolol (P0778, ≥98%), and carvedilol (C3993, ≥98%) were purchased from Sigma-Aldrich (St. Louis, MO, USA). Norepinephrine (N661038, ≥98%), (*R*)-salbutamol (A511485, ≥98%), (*S*)-salbutamol (A511490, ≥98%), and carazolol (C175810, ≥98%) were obtained from Toronto Research Chemicals (Vaughan, ON, Canada). Formoterol (F0372000, ≥98%) and alprenolol (A0360000, ≥98%) were obtained from the European Directorate for the Quality of Medicines & HealthCare (EDQM, Strasbourg, France). Orciprenaline (HY-B1276, ≥99%) and carmoterol (HY-12463, ≥98%) were purchased from MedChemExpress (Princeton, NJ, USA). Isoprenaline, epinephrine, dobutamine, pindolol, (*R*)-salbutamol, (*S*)-salbutamol, formoterol, alprenolol, and orciprenaline were dissolved in water to prepare stock solutions. All remaining ligands were dissolved in dimethyl sulfoxide (DMSO) to prepare stock solutions. Working solutions were prepared by dilution into water immediately before use. The final DMSO concentration in all assays was ≤1% (v/v).

### Expression and Purification of β_1_AR, Nanobody Variants and Mini-Gs β_1_AR

The gene encoding the thermostabilized turkey β_1_AR from *Meleagris gallopavo* was generated as previously described.^17,29,47^ The construct comprises residues 45–243 and 272–367, corresponding to N- and C-terminal truncations and deletion of most of ICL3. In addition, the construct contains five thermostabilizing mutations (R68S, M90 V, E130W, F327A, and F338M), one mutation to improve protein expression (C116L), one mutation to prevent potential palmitoylation (C358A), and one mutation designed to enhance active-state nanobody binding (R284K). The construct was cloned into the baculovirus transfer vector pFastBac1 (Thermo Fisher Scientific, Waltham, MA) for expression in Sf9 insect cells (Invitrogen, Carlsbad, CA) cultured in Sf-900 III SFM medium (Gibco, Waltham, MA). Recombinant baculovirus was generated using the Bac-to-Bac baculovirus expression system (Thermo Fisher Scientific). The receptor construct incorporated purification tags at both the N- and C-termini, each followed by a protease cleavage site. To facilitate site-specific bimane labeling for monitoring TM6 movement, native reactive cysteines within this thermostabilized β_1_AR were mutated to nonreactive residues (C85 V, C116L, and C344S), and a single cysteine was introduced at position 282 (A282C).^38^

To purify the receptor, Sf9 cell pellets were harvested 48 h postinfection and resuspended in lysis buffer (20 mM Tris-HCl, 1 mM EDTA, pH 8.0) supplemented with a protease inhibitor cocktail (Roche, Basel, Switzerland). Cells were lysed by two passes through a high-pressure microfluidizer at 9,000 psi. The lysate was clarified by centrifugation at 9,000*g* for 20 min at 4 °C, and the supernatant was subjected to ultracentrifugation at 200,000*g* for 1 h at 4 °C to pellet the membrane fraction. The membrane pellet was resuspended in wash buffer (20 mM Tris-HCl, 1 mM EDTA, pH 8.0) and repelleted by ultracentrifugation (200,000*g*, 1 h, 4 °C). This wash step was repeated once with wash buffer containing 0.2 mM EDTA. The washed membranes were resuspended in solubilization buffer (20 mM Tris-HCl, 700 mM NaCl, 6 mM imidazole, 3% (w/v) *n*-dodecyl-β-D-maltopyranoside (DDM, Anatrace, Maumee, OH), pH 8.0) and solubilized for 1 h at 4 °C with gentle agitation. Insoluble material was removed by ultracentrifugation at 100,000*g* for 1 h at 4 °C, and the clarified extract was filtered through a 0.22 μm membrane.

The filtrate was loaded onto a 5 mL HisTrap TALON Crude column (Cytiva, Marlborough, MA) pre-equilibrated with binding buffer (20 mM Tris-HCl, 350 mM NaCl, 3 mM imidazole, 0.05% (w/v) DDM, pH 8.0). The column was washed with 10 column volumes (CV) of binding buffer, and bound protein was eluted with a linear gradient to elution buffer (20 mM Tris-HCl, 350 mM NaCl, 350 mM imidazole, 0.05% (w/v) DDM, pH 8.0) over 3 CV. Eluted fractions were pooled, concentrated to approximately 1 mg/mL, and desalted into binding buffer using a desalting column. Protein purity was confirmed to be >95% by SDS-PAGE prior to subsequent experiments.

### Nanobody Variants

All nanobody variants (Nb80, Nb6B9, Nb71, Nb60, and Nb6B9.1–3) were cloned into pET24­(+) with an N-terminal signal sequence and a C-terminal 6 × His tag and transformed into *Escherichia coli* BL21­(DE3) cells (Agilent, Santa Clara, CA). Cultures were grown in LB broth supplemented with 50 μg/mL kanamycin (Sigma-Aldrich) at 37 °C to an OD_600_ of 0.6–0.8, then induced with 1 mM IPTG (Formedium, Hunstanton, UK) and incubated overnight at 25 °C with shaking at 200 rpm. Cell pellets were resuspended in lysis buffer (20 mM Tris-HCl, 100 mM NaCl, 10 mM imidazole, 10% (v/v) glycerol, pH 8.0) supplemented with a protease inhibitor cocktail, benzonase nuclease (25 U mL^–1^, Millipore, Burlington, MA), and 1 mM DTT. Cells were lysed via four passes through a high-pressure microfluidizer at 18,000 psi. The lysate was clarified by centrifugation at 38,000*g* for 30 min at 4 °C and filtered through a 0.22 μm membrane. The clarified lysate was loaded onto a 5 mL HisTrap TALON Crude column pre-equilibrated with binding buffer (20 mM Tris-HCl, 100 mM NaCl, 10 mM imidazole, 10% (v/v) glycerol, pH 8.0). The resin was washed with 20 CV of binding buffer, followed by 10 CV of high-salt wash buffer (20 mM Tris-HCl, 500 mM NaCl, 30 mM imidazole, 10% (v/v) glycerol, pH 8.0) and an additional 5 CV of binding buffer. Protein was eluted over 3 CV using an elution buffer containing 500 mM imidazole. Eluted fractions were pooled, concentrated to ∼0.5 mg/mL, and desalted into storage buffer (20 mM Tris-HCl, pH 8.0, 100 mM NaCl, 10% (v/v) glycerol). Final protein purity was confirmed to be >95% homogeneous by SDS-PAGE prior to subsequent experiments.

### Mini-Gs

The engineered mini-Gs construct was cloned into the expression vector pET24­(+), incorporating a C-terminal TEV protease cleavage site followed by a 6 × His tag. The plasmid was expressed in *E. coli* BL21­(DE3) strain. Culture growth and expression were carried out as described for the nanobody variants, except that induction was performed with 50 μM IPTG. Cell pellets were resuspended in mini-Gs lysis buffer (40 mM HEPES, 100 mM NaCl, 10 mM imidazole, 5 mM MgCl_2_, 10% (v/v) glycerol, pH 7.5) supplemented with a protease inhibitor cocktail, benzonase nuclease, and 1 mM DTT, and lysed similarly using the microfluidizer. The clarified lysate was applied to a 5 mL HisTrap TALON Crude column pre-equilibrated with mini-Gs binding buffer (40 mM HEPES, 100 mM NaCl, 10 mM imidazole, 1 mM MgCl_2_, 10% (v/v) glycerol, 50 μM GDP, pH 7.5). The resin was washed sequentially with 20 CV of binding buffer containing 50 μM GDP, 10 CV of high-salt wash buffer (20 mM HEPES, 500 mM NaCl, 30 mM imidazole, 1 mM MgCl_2_, 10% (v/v) glycerol, 50 μM GDP, pH 7.5), and an additional 5 CV of binding buffer with 50 μM GDP. Protein was eluted over 3 CV with elution buffer (20 mM HEPES, 100 mM NaCl, 500 mM imidazole, 1 mM MgCl_2_, 10% (v/v) glycerol, 50 μM GDP, pH 7.5). Eluted protein was concentrated and desalted into storage buffer (20 mM HEPES, 100 mM NaCl, 10 μM GDP, 1 mM MgCl_2_, 10% (v/v) glycerol, pH 7.5). The 6 × His tag was removed via overnight cleavage at 4 °C with TEV protease (20:1 mini-Gs: TEV mass ratio), and the cleaved protein was collected from the flow-through of a 1 mL TALON column. The protein was concentrated to approximately 1 mg/mL, with final purity confirmed at > 95% homogeneity by SDS-PAGE.

### Native Mass Spectrometry Analysis of Complex Formation

Receptor–nanobody complexes under ligand treatment were characterized by nMS using a Q-Exactive UHMR Orbitrap mass spectrometer (Thermo Fisher Scientific). The species analyzed included β_1_AR monomer and its complexes with Nb80, Nb60, Nb6B9, Nb6B9 variants, Nb71 and mini-Gs. Prior to analysis, purified proteins were buffer-exchanged into 200 mM ammonium acetate containing 2× the critical micelle concentration of custom-synthesized detergent G1 using Zeba Spin desalting columns (Thermo Fisher Scientific). The G1 detergent was provided by the Synthesis Core Facility, Institute of Biological Chemistry, Academia Sinica (Taipei, Taiwan).

Unless otherwise specified, all ligand-treated samples were prepared by incubating purified β_1_AR with each ligand at a final concentration of 100 μM for 30 min on ice prior to complex assembly. β_1_AR and nanobodies were mixed at a 1:1 molar ratio for all complex-formation experiments, except for nanobody variants or mini-Gs titration experiments used to determine maximum complex-formation levels. Ions were generated by nanoelectrospray ionization using in-house-prepared, gold-coated borosilicate glass capillaries at an applied voltage of 1.1 kV. In-source collision-induced dissociation was applied at 30 eV, and in-source trapping was set to −100 V to aid desolvation. A higher-energy collisional dissociation energy of 5 V was applied to enhance protein signal release from proteomicelles. Additional instrument parameters were as follows: capillary temperature, 200 °C; S-lens RF level, 200; ion injection time (fixed), 100 ms; resolution, 12,500.

### Bimane Labeling and Fluorescence Spectroscopy Assays

Purified β_1_AR protein engineered with a bimane labeling site was incubated with monobromobimane (Thermo Fisher Scientific) at a 1:5 protein-to-dye molar ratio in protein buffer overnight at 4 °C. The labeled protein at 1 μM was subsequently buffer-exchanged into 200 mM ammonium acetate containing 2× CMC of G1 detergent. Successful labeling was validated via nMS analysis. For fluorescence measurements, the labeled protein was transferred to a 384-well black microplate (Greiner Bio-One, Kremsmünster, Austria). Basal background (untreated) and ligand-treated samples (final ligand concentration of 100 μM) were prepared. The plate was centrifuged at 1000*g* for 2 min and incubated in the dark at 4 °C for 1 h. Fluorescence spectra were recorded using a Tecan Spark microplate reader (Tecan Group Ltd., Männedorf, Switzerland) with an excitation wavelength of 373 nm (bandwidth 5 nm) and emission scanned from 400 to 600 nm (bandwidth 10 nm).

### Statistical Analysis

#### Quantification of the Receptor-Nanobody Complex

Mass spectra were analyzed using Xcalibur Qual Browser (version 4.4.16.14, Thermo Fisher Scientific). The relative abundances of receptor monomer and receptor-nanobody complexes were quantified by UniDec (version 8.1.0). The complex formation level (%) was calculated from the deconvoluted peak intensities as follows

1 $$\mathrm{Co}mplex\;Formation\;Level\;\left(\%\right)=\frac{I_{receptor‐nanobodycomplex}}{I_{monomer}+I_{receptor‐nanobodycomplex}}\times 100$$

where *I*_receptor‑nanobodycomplex_ and *I*_monomer_ denote the summed signal intensities of the receptor-nanobody complex and free receptor monomer, respectively. All measurements were performed in at least 3 independent biological replicates. Data are presented as mean ± SD.

#### Correlation between nMS Complex Formation and Cellular cAMP Signaling

Cellular cAMP accumulation values for ten ligands (isoprenaline, adrenaline, noradrenaline, formoterol, racemic salbutamol, dobutamine, orciprenaline [reported as metaproterenol], carvedilol, pindolol, and alprenolol) were obtained from a previously reported pharmacological characterization of the β6–m23 thermostabilized turkey β_1_-adrenoceptor, expressed as a percentage of the maximal isoprenaline response in ^3^H-cAMP accumulation assays.^30^ To validate our method, these cellular cAMP response values were paired with our corresponding nMS-derived complex formation levels for each ligand and evaluated by Pearson correlation analysis. The analysis reported the correlation coefficient (*r*), coefficient of determination (*R* ^2^), and two-tailed *P* value, with *P* < 0.05 considered statistically significant. All statistical analyses were performed using GraphPad Prism 10 (GraphPad Software, Boston, MA).

#### Dose–Response Analysis on Salbutamol Enantiomers

Dose–response data for (*R*)- and (*S*)-salbutamol were analyzed by nonlinear regression using the log­[agonist] vs response, variable-slope (four-parameter) model

2 $$Y=Bottom+\frac{\left(Top-Bottom\right)}{1+10^{\left(\log{EC}_{50}-X\right)\times HillSlope}}$$

where *X* is the log_10_-transformed agonist concentration and *Y* is the normalized complex formation level (%). EC_50_ values were obtained from the fitted Log EC_50_ parameter (EC_50_ = 10^LogEC_50_^), and *E*_max_ was defined as the span (Top – Bottom) of the fitted curve. The statistical analyses were performed using GraphPad Prism 10.

#### Bimane Fluorescence Analysis

To analyze the fluorescence response, emission fluorescence intensities from 400 to 600 nm for each sample (*n* = 3) were normalized to the maximum mean fluorescence value of the vehicle control group. Data plotting and statistical analyses were performed using GraphPad Prism 10. Unless otherwise stated, data are presented as mean ± SD from *n* = 3 independent biological replicates.

#### Nb6B9 Chimeric Variants Comparison

To compare the activity of these chimeric variants relative to the parental Nb6B9, the complex formation levels for each variant were expressed as the difference from the mean complex formation level of the parental Nb6B9 measured under the same ligand condition. All data are presented as mean ± SEM from *n* = 3 independent biological replicates. Statistical analyses were performed using GraphPad Prism 10 (GraphPad Software, Boston, MA).

#### Structural Analysis

Three-dimensional structures of thermostabilized β_1_AR in the inactive state (PDB: 4GPO) and active state (PDB: 6H7L) were visualized and analyzed. The structural model of the inactive-state specific nanobody Nb60 (PDB: 5JQH), along with those of active-state binding partners, mini-Gs (PDB: 5G53), Nb80 (PDB: 6H7J), Nb6B9 (PDB: 6H7L and 6H7M), and Nb71 (PDB: 6MXT), were used for structural comparisons. All visualization and analyses were performed using PyMOL Molecular Graphics System (version 3.0, Schrödinger LLC, New York, NY) and UCSF ChimeraX 1.12 (University of California, San Francisco, CA). Structural superpositions were performed using the cealign function in PyMOL, and structural similarity was quantified by root-mean-square deviation of Cα atoms. These published structural models were utilized for structural comparison and figure generation throughout the manuscript.

#### Sequence Alignment

Sequences of Nb6B9, Nb80, Nb60 and Nb71, were retrieved from their corresponding PDB entries. Multiple sequence alignment was performed using Clustal Omega (EMBL-EBI, Hinxton, Cambridgeshire, UK) and visualized using ESPript 3.0 (Institut de Biologie et Chimie des Protéines, Lyon, France).^48,49^

## Supplementary Material





## Notes

Additional experimental details, including workflow schemes, ligand chemical structures, representative mass spectra, and quantitative complex-formation tables (DOCX)
Table of detected mass species (XLSX)

## Abbreviations

ADR: adrenaline
ALP: alprenolol
β_1_AR: β_1_-adrenergic receptor
CARAZ: carazolol
CARM: carmoterol
CARV: carvedilol
CDR: complementarity-determining region
CMC: critical micelle concentration
cryo-EM: cryogenic electron microscopy
CV: column volume
DDM: n-dodecyl-β-D-maltopyranoside
DMSO: dimethyl sulfoxide
DOB: dobutamine
FORM: formoterol
GDP: guanosine diphosphate
GPCR: G protein-coupled receptor
HDX-MS: hydrogen–deuterium exchange mass spectrometry
ICL: intracellular loop
IPTG: isopropyl β-D-1-thiogalactopyranoside
ISO: isoprenaline
mBBr: monobromobimane
NMR: nuclear magnetic resonance
nMS: native mass spectrometry
NOADR: noradrenaline
ORCI: orciprenaline
PIN: pindolol
(*R*)-SALB: (*R*)-salbutamol
(*S*)-SALB: (*S*)-salbutamol
SD: standard deviation
SEM: standard error of the mean
TEV: tobacco etch virus protease.
