# Supplementary material for: Native Mass Spectrometry Reveals Ligand-Specific Conformational Heterogeneity of the β1‑Adrenergic Receptor Using Nanobody Probes
Source: Anal Chem. 2026 Sep 26;98(40):29378–88. doi: 10.1021/acs.analchem.6c04571 (PMC13670896; doi:10.1021/acs.analchem.6c04571)
Supplement: Supplementary file 1 [file ac6c04571_si_001.docx]

**Supporting Information**

**Native Mass Spectrometry Reveals Ligand-Specific Conformational Heterogeneity of the β_1_-Adrenergic Receptor Using Nanobody Probes**

**Yi-Quan Wang^1,§^, Guan-Ting Lian^1,2,3,§^, Yi-An Chen^1^, Rong Chen^1,2^, Peng-Yi Wu^1,2^, Hsiang-Hui Peng^1^, Hsin-Yung Yen^1,2,*^**

^1^ Institute of Biological Chemistry, Academia Sinica, Taipei 115201, Taiwan.

^2^ Institute of Biochemical Sciences, National Taiwan University, Taipei 106319, Taiwan.

^3^ Chemical Biology and Molecular Biophysics Program, Taiwan International Graduate Program, Academia Sinica, Taipei 115201, Taiwan.

^§^ These authors contributed equally: Yi-Quan Wang, Guan-Ting Lian

*Correspondence: [hsinyungyen@gate.sinica.edu.tw](mailto:hsinyungyen@gate.sinica.edu.tw)

**Table of Contents**

Table S1. Ligand-dependent β1AR complex formation with mini-Gs and nanobodies quantified by nMS.

Table S2. Ligand-dependent β1AR complex formation with Nb6B9 and its CDR chimeras.

Table S3. Dose-dependent β1AR-Nb6B9 complex formation induced by salbutamol enantiomers.

Figure S1. Experimental workflow of the nanobody-based nMS platform.

Figure S2. Chemical structures of β₁AR ligands used in this study.

Figure S3. Representative nMS spectra of β₁AR-Nb80 complex formation across the ligand panel.

Figure S4. Representative nMS spectra of β₁AR-Nb71 complex formation across the ligand panel.

Figure S5. Representative nMS spectra of β₁AR-Nb6B9 complex formation across the ligand panel.

Figure S6. Representative nMS spectra of β₁AR-Nb60 complex formation.

Figure S7. Native MS characterization of β1AR-mini-Gs complex formation and its correlation with cAMP signaling.

Figure S8. Amino acid sequence alignment of nanobodies and the design of Nb6B9 CDR chimeric variants.

Figure S9. Construct design and verification of bimane-labeled β₁AR.

Figure S10. Native MS characterization of β₁AR-Nb6B9.1 complex formation across the ligand panel.

Figure S11. Native MS characterization of β₁AR-Nb6B9.2 complex formation across the ligand panel.

Figure S12. Native MS characterization of β₁AR-Nb6B9.3 complex formation across the ligand panel. Figure S13. Structural comparison of salbutamol enantiomers in the β₁AR orthosteric binding pocket.

References

**Table S1. Ligand-dependent β_1_AR complex formation with mini-Gs and nanobodies quantified by nMS.**

| **Ligands** | **mini-Gs (%)** | **Nb71 (%)** | **Nb6B9 (%)** | **Nb80 (%)** |
| --- | --- | --- | --- | --- |
| **Apo** | 0.0 ± 0.0 | 0.0 ± 0.0 | 8.2 ± 5.1 | 0.0 ± 0.0 |
| **Isoprenaline** | 77.3 ± 5.2 | 82.2 ± 1.9 | 86.2 ± 7.8 | 78.1 ± 2.3 |
| **Epinephrine** | 70.4 ± 7.6 | 78.0 ± 10.0 | 88.8 ± 3.8 | 71.2 ± 9.6 |
| **Norepinephrine** | 72.1 ± 10.2 | 74.0 ± 4.6 | 89.7 ± 2.5 | 71.1 ± 7.8 |
| **Formoterol** | 54.0 ± 16.3 | 72.4 ± 7.4 | 85.8 ± 8.9 | 67.3 ± 8.3 |
| **Orciprenaline** | 76.2 ± 7.8 | 64.8 ± 4.1 | 78.3 ± 3.3 | 51.2 ± 3.0 |
| **(*R*)-Salbutamol** | 47.3 ± 10.3 | 62.9 ± 2.0 | 77.4 ± 1.0 | 48.0 ± 8.4 |
| **Dobutamine** | 6.7 ± 6.7 | 10.2 ± 5.6 | 52.3 ± 11.1 | 9.7 ± 2.9 |
| **Pindolol** | 0.0 ± 0.0 | 3.6 ± 3.1 | 45.7 ± 11.6 | 5.2 ± 1.2 |
| **Alprenolol** | 1.6 ± 2.7 | 3.8 ± 3.4 | 49.6 ± 4.1 | 10.4 ± 7.9 |
| **Carvedilol** | 4.0 ± 7.0 | 0.0 ± 0.0 | 13.8 ± 6.1 | 0.0 ± 0.0 |
| **Carazolol** | - | - | 23.3 ± 4.9 | 1.7 ± 3.0 |
| **Carmoterol** | - | - | 82.8 ± 1.0 | 47.0 ± 12.0 |
| **(*S*)-Salbutamol** | - | - | 51.9 ± 3.9 | 10.8 ± 4.4 |

Values represent the percentage of β_1_AR in complex with mini-Gs, Nb71, Nb6B9, or Nb80 under each ligand treatment. Data are presented as mean ± SD from at least three independent measurements. Dashes indicate conditions not assessed.

**Table S2. Ligand-dependent β_1_AR complex formation with Nb6B9 and its CDR chimeras.**

| **Ligands** | **Nb6B9 (%)** | **Nb6B9.1 (%)** | **Nb6B9.2 (%)** | **Nb6B9.3 (%)** |
| --- | --- | --- | --- | --- |
| **Apo** | 8.2 ± 5.1 | 0.0 ± 0.0 | 20.2 ± 11.6 | 0.0 ± 0.0 |
| **Isoprenaline** | 86.2 ± 7.8 | 82.7 ± 8.2 | 88.9 ± 5.1 | 78.6 ± 6.7 |
| **(*R*)-Salbutamol** | 77.4 ± 1.0 | 67.3 ± 10.5 | 85.8 ± 7.3 | 58.7 ± 8.6 |
| **Dobutamine** | 52.3 ± 11.1 | 31.1 ± 8.2 | 75.3 ± 8.6 | 18.0 ± 2.4 |
| **Pindolol** | 45.7 ± 11.6 | 28.7 ± 4.9 | 71.4 ± 1.0 | 12.6 ± 4.4 |
| **Alprenolol** | 49.6 ± 4.1 | 30.5 ± 2.5 | 74.8 ± 4.6 | 13.2 ± 1.7 |
| **Carvedilol** | 13.8 ± 6.1 | 5.4 ± 3.6 | 37.5 ± 6.5 | 0.0 ± 0.0 |
| **Carazolol** | 23.3 ± 4.9 | 17.9 ± 2.4 | 62.0 ± 4.5 | 22.2 ± 9.2 |
| **(*S*)-Salbutamol** | 51.9 ± 3.9 | 30.6 ± 9.2 | 71.9 ± 12.2 | 7.8 ± 3.1 |

Values represent the percentage of β_1_AR in complex with Nb6B9 and its CDR chimeric variants under each ligand treatment. Data are presented as mean ± SD from at least three independent measurements.

**Table S3. Dose-dependent β_1_AR-Nb6B9 complex formation induced by salbutamol enantiomers.**

| **Drug Conc. (μM)** | **(*R*)-Salbutamol (%)** | **(*S*)-Salbutamol (%)** |
| --- | --- | --- |
| **0.625** | 7.3 ± 1.4 | 0.0 ± 0.0 |
| **1.25** | 5.6 ± 0.3 | 1.9 ± 3.3 |
| **2.5** | 17.2 ± 3.9 | 1.8 ± 3.1 |
| **5** | 77.4 ± 1.0 | 0.0 ± 0.0 |
| **10** | 49.3 ± 7.2 | 11.2 ± 4.2 |
| **20** | 61.9 ± 2.6 | 11.3 ± 4.3 |
| **50** | 63.0 ± 2.8 | 27.2 ± 7.0 |
| **100** | 77.4 ± 1.0 | 50.4 ± 1.4 |
| **200** | 83.6 ± 3.3 | 49.2 ± 5.0 |

Values represent the percentage of β1AR-Nb6B9 complex formation following treatment with (*R*)- or (*S*)-salbutamol at increasing concentrations. Data are shown as mean ± SD from at least three independent measurements.

**Figure S1. Experimental workflow of the nanobody-based nMS platform.** Purified β₁AR was incubated with selected ligands and intracellular binding partners, including active-state stabilizing nanobodies (Nb80, Nb6B9, and Nb71), the inactive-state stabilizing nanobody Nb60, and mini-Gs. Ligand-stabilized receptor-nanobody assemblies were introduced into the gas phase utilizing nano-electrospray ionization. Detergent micelles were subsequently removed via collisional activation, enabling the precise mass measurement of intact receptor monomers and receptor-partner complexes within the mass spectrometer. Finally, the relative abundance of complex formation was quantified to evaluate ligand-dependent binding efficiency across the panel of conformational nanobody probes.

**Figure S2.** **Chemical structures of β₁AR ligands used in this study.** Chemical structures and abbreviations of the evaluated β₁AR ligands: (1) noradrenaline (NOADR), (2) adrenaline (ADR), (3) isoprenaline (ISO), (4) (*R*)-salbutamol ((*R*)-SALB), (5) orciprenaline (ORCI), (6) formoterol (FORM), (7) dobutamine (DOB), (8) alprenolol (ALP), (9) pindolol (PIN), (10) carvedilol (CARV), (11) (*S*)-salbutamol ((*S*)-SALB), (12) carmoterol (CARM), (13) carazolol (CARAZ). Compound numbers correspond to the ligand identifiers used throughout the main text, figures, and supplementary tables. Chiral centers are denoted by asterisksas (*).

**Figure S3.** **Representative nMS spectra of β₁AR-Nb80 complex formation across the ligand panel.** β₁AR was incubated with Nb80 in the absence of ligand or in the presence of the indicated ligands, followed by native MS analysis. Charge-state distributions corresponding to free Nb80, monomeric β₁AR, and β₁AR-Nb80 complex are annotated in orange, blue, and dark yellow, respectively.

**Figure S4.** **Representative nMS spectra of β₁AR-Nb71 complex formation across the ligand panel.** β₁AR was incubated with Nb71 in the absence of ligand or in the presence of the indicated ligands, followed by native MS analysis. Charge-state series corresponding to free Nb71, β₁AR monomer, and β₁AR-Nb71 complex are annotated in light green, blue, and dark green, respectively.

**Figure S5. Representative nMS spectra of β₁AR-Nb6B9 complex formation across the ligand panel.** β₁AR was incubated with Nb6B9 in the absence of ligand or in the presence of the indicated ligands, followed by native MS analysis. Peaks corresponding to free Nb6B9, β₁AR monomer, and β₁AR-Nb6B9 complex in different charge states are annotated in salmon, blue, and magenta red, respectively.

**Figure S6.** **Representative nMS spectra of β₁AR-Nb60 complex formation. (a-b)** β₁AR complex formation with Nb60 in the absence of ligand (**a**) or in the presence of carazolol (**b**) and isoprenaline (**c**). Charge-state series corresponding to free Nb60, β₁AR monomer, and β₁AR–Nb60 complex are annotated in light taupe, blue, and tan-orange, respectively.

**Figure S7.** **Native** **MS characterization of β_1_AR-mini-Gs complex formation and its correlation with cAMP signaling.** (**a**) Representative nMS spectra of β₁AR in complex with mini-Gs in the absence of ligand or in the presence of the indicated ligands. Charge-state series corresponding to free mini-Gs, β₁AR monomer, and β₁AR-mini-Gs complex are annotated in light lavender, blue, and purple, respectively. (**b**) Correlation plots comparing cellular cAMP response, adapted from literature data^1^ against nMS-derived complex formation levels for β₁AR-mini-Gs assembly.

**Figure S8.** **Amino acid sequence alignment of nanobodies and the design of Nb6B9 CDR chimeric variants.** (**a**) Multiple sequence alignment of Nb80, Nb6B9, Nb71, and Nb60. Framework regions (FR1–FR4) and complementarity-determining regions (CDR1-CDR3) are annotated above the sequences. Residues conserved across all four nanobodies are shaded in red blocks; partially conserved positions are shown in red text. Secondary structures are assigned based on the Nb80 crystal structure.^2^ Multiple sequence alignment was performed using Clustal Omega and visualized using ESPript 3.0.^3, 4^ (**b**) CDR Sequences comparison between Nb6B9 and Nb80. Nb6B9.1 contains CDR1 substitutions A30S, L31I and I33T, Nb6B9.2 contains the CDR2 substitution T56S, and Nb6B9.3 contains CDR3 substitutions F100Y, I103V, I104L and D106E.

**Figure S9.** **Construct** **design and verification of bimane-labeled β₁AR.** (**a**) Snake-plot representation of the engineered β₁AR construct utilized for bimane fluorescence assays, generated with Protter.^5^ The position of the introduced cysteine residue (Cys^6.27^) near the cytoplasmic end of TM6 is highlighted for mBBr labeling as described in Experimental section. **(b)** Native MS spectra confirming mBBr labeling of β₁AR. The labeled receptor exhibits a uniform mass shift of approximately 192 Da compared to the unlabeled receptor, demonstrating the site-specific incorporation of a single bimane moiety.

**Figure S10.** **Native MS characterization of β₁AR-Nb6B9.1 complex formation across the ligand panel.** Representative nMS spectra are shown for β₁AR-Nb6B9.1 complex formation in the absence of ligand or in the presence of the indicated ligands. Charge-state series corresponding to free Nb6B9.1, β₁AR monomer, and β₁AR-Nb6B9.1 complex are annotated in yellow, blue, and orange, respectively.

**Figure S11.** **Native MS characterization of β₁AR-Nb6B9.2 complex formation across the ligand panel.** β₁AR was incubated with the Nb6B9.2 chimeric variant either in the absence of ligand or in the presence of the indicated ligands. MS analysis revealed charge-state distributions corresponding to free Nb6B9.2, β₁AR monomer, and β₁AR-Nb6B9.2 complex are annotated in pink, blue, and purple, respectively.

**Figure S12.** **Native MS characterization of β₁AR-Nb6B9.3 complex formation across the ligand panel.** Representative nMS spectra are shown for β₁AR-Nb6B9.3 complex formation in the absence of ligand or in the presence of the indicated ligands. Charge-state distributions corresponding to free Nb6B9.3, β₁AR monomer, and β₁AR-Nb6B9.3 complex are annotated in tan, blue, and dark brown, respectively.

**Figure S13.** **Structural comparison of salbutamol enantiomers in the β₁AR orthosteric binding pocket.** (**a**) Close-up view of the β₁AR orthosteric binding pocket bound to (*R*)-salbutamol (PDB: 6H7M).^6^ Polar interactions between (*R*)-salbutamol and key ligand-binding residues are indicated by dashed yellow lines, with distances labeled in angstroms (Å). Residues are annotated using the combined Ballesteros–Weinstein/GPCRdb generic numbering scheme.^7^ (**b**) Structural comparison of (*R*)-salbutamol and simulated (*S*)-salbutamol poses relative to D121^3.32x32^ and N329^7.39x38^. The inverted configuration at the β-hydroxyl-bearing chiral center of the (*S*)-enantiomer disrupts the critical polar interaction network, potentially explaining its altered active-state stabilization.

**References**

(1) Baker, J. G.; Proudman, R. G.; Tate, C. G. The pharmacological effects of the thermostabilising (m23) mutations and intra and extracellular (β36) deletions essential for crystallisation of the turkey β-adrenoceptor. *Naunyn Schmiedebergs Arch Pharmacol* **2011**, *384* (1), 71-91. DOI: 10.1007/s00210-011-0648-4

(2) Rasmussen, S. G.; Choi, H. J.; Fung, J. J.; Pardon, E.; Casarosa, P.; Chae, P. S.; Devree, B. T.; Rosenbaum, D. M.; Thian, F. S.; Kobilka, T. S.; Schnapp, A.; Konetzki, I.; Sunahara, R. K.; Gellman, S. H.; Pautsch, A.; Steyaert, J.; Weis, W. I.; Kobilka, B. K. Structure of a nanobody-stabilized active state of the β(2) adrenoceptor. *Nature* **2011**, *469* (7329), 175-180. DOI: 10.1038/nature09648

(3) Madeira, F.; Madhusoodanan, N.; Lee, J.; Eusebi, A.; Niewielska, A.; Tivey, A. R. N.; Lopez, R.; Butcher, S. The EMBL-EBI Job Dispatcher sequence analysis tools framework in 2024. *Nucleic Acids Res* **2024**, *52* (W1), W521-w525. DOI: 10.1093/nar/gkae241

(4) Robert, X.; Gouet, P. Deciphering key features in protein structures with the new ENDscript server. *Nucleic Acids Res* **2014**, *42* (Web Server issue), W320-324. DOI: 10.1093/nar/gku316

(5) Omasits, U.; Ahrens, C. H.; Müller, S.; Wollscheid, B. Protter: interactive protein feature visualization and integration with experimental proteomic data. *Bioinformatics* **2014**, *30* (6), 884-886. DOI: 10.1093/bioinformatics/btt607

(6) Warne, T.; Edwards, P. C.; Doré, A. S.; Leslie, A. G. W.; Tate, C. G. Molecular basis for high-affinity agonist binding in GPCRs. *Science* **2019**, *364* (6442), 775-778. DOI: 10.1126/science.aau5595

(7) Isberg, V.; de Graaf, C.; Bortolato, A.; Cherezov, V.; Katritch, V.; Marshall, F. H.; Mordalski, S.; Pin, J. P.; Stevens, R. C.; Vriend, G.; Gloriam, D. E. Generic GPCR residue numbers - aligning topology maps while minding the gaps. *Trends Pharmacol Sci* **2015**, *36* (1), 22-31. DOI: 10.1016/j.tips.2014.11.001
